# Does Robot Assisted Laparoscopy (RAL) Have an Advantage in Preservation of Ovarian Reserve in Endometriosis Surgery? Comparison of Single-Port Access (SPA) RAL and SPA Laparoscopy

**DOI:** 10.3390/jcm12144673

**Published:** 2023-07-14

**Authors:** Jun-Hyeok Kang, Chi-Son Chang, Joseph J. Noh, Tae-Joong Kim

**Affiliations:** 1 Department of Obstetrics and Gynecology, Uijeongbu Eulji Medical Center, Eulji University School of Medicine, Uijeongbu-si 11759, Gyeonggi-do, Republic of Korea; junhyuck1985@gmail.com; 2 Department of Obstetrics and Gynecology, Chung-Ang University Gwang-Myung Hospital, Chung-Ang University College of Medicine, Gwangmyeong-si 14353, Gyeonggi-do, Republic of Korea; cschang.med@gmail.com; 3 Department of Obstetrics and Gynecology, Samsung Medical Center, Sungkyunkwan University School of Medicine, Seoul 06351, Republic of Korea; joseph.noh@samsung.com

**Keywords:** robot assisted laparoscopy, single-port access laparoscopy, endometriosis, ovarian reserve, AMH

## Abstract

The purpose of this study was to compare single-port access (SPA) laparoscopy and SPA robot assisted laparoscopy (RAL) for endometriosis with respect to ovarian reserve preservation and surgical outcomes. Clinical factors affecting any reduction in ovarian function after surgery were also evaluated. Patients with endometriosis who underwent SPA laparoscopy (n = 87) or RAL (n = 78) were retrospectively reviewed. Patients’ baseline characteristics, including the severity of endometriosis and surgical outcomes including surgical complexity, were collected. To assess the preservation of ovarian reserve after surgery, serum anti-Müllerian hormone (AMH) levels before surgery, at two weeks, and at three months after surgery were collected. Age, ovarian cyst size, location of cyst, complexity of surgery, and the severity of endometriosis were associated with the reduction in AMH levels after surgery. The severity of endometriosis was higher in the RAL group than in the SPA group. There were no significant differences in other clinical baseline characteristics, including preoperative AMH levels. For surgical outcomes, radical surgery was more frequently performed in the RAL group. In univariate and multivariate linear regression analyses, age, ovarian cyst size, location of cyst, complexity of surgery, and the severity of endometriosis were associated with the reduction in AMH levels after surgery. Incorporating surgical approaches and risk factors for postoperative ovarian function decrease, RAL was more beneficial than SPA laparoscopy for the preservation of ovarian reserve in patients with mild endometriosis (stage I/II) (postoperative 3 month AMH reduction rate (%), SPA laparoscopy vs. RAL, 33.51 ± 19.98 vs. 23.58 ± 14.98, *p* = 0.04) and in patients who underwent non-complex surgery (postoperative 3 month AMH reduction rate (%), SPA laparoscopy vs. RAL, 37.89 ± 22.37 vs. 22.37 ± 17.49, *p* = 0.022). SPA RAL may have advantages over SPA laparoscopy in ovarian function preservation, especially in patients with mild endometriosis and patients who have undergone a non-complex surgery.

## 1. Introduction

Endometriosis is a common gynecological disorder defined as the presence of endometrial glands and stroma outside the uterine cavity, such as in the ovary and peritoneum, with an estimated incidence of 10% in the general population [1]. The infiltrative nature of this disease can cause fibrosis and adhesions between pelvic organs, causing distortion of the normal pelvic anatomy [2]. It also invades the ovarian cortex and negatively affects ovarian reserve [3]. Therefore, it can lead to problems ranging from chronic pelvic pain to infertility, thus decreasing the quality of life of patients [4,5].

Complete surgical resection of all visible lesions and restoration of distorted anatomical structures using laparoscopy is the gold standard treatment for endometriosis when medical treatment fails [6,7]. Considering that the majority of patients with endometriosis are reproductive-aged women, it is important to preserve ovarian reserve after surgery as well as to restore their normal pelvic structure through radical resection of endometriosis lesions [8]. Recently, single-port access (SPA) laparoscopy has gained popularity among young women because of its cosmetic benefits and decreased postoperative pain [9,10]. It is gradually replacing multiport laparoscopy in benign gynecologic surgery. However, SPA laparoscopy is difficult and challenging due to the surgeon’s less ergonomic positions and frequent collisions among instruments. These difficulties become more challenging in endometriosis surgery, in which it is often required to perform adhesiolysis of destructed dense tissue and stripping of ovarian surface. Furthermore, the rough surgical action induced by such technical limitations has been regarded as a major disadvantage of SPA laparoscopy for endometriosis surgery, especially for the preservation of ovarian function [11]. On the other hand, robot assisted laparoscopy (RAL), which is considered as the most advanced technology for minimally invasive surgery (MIS), is emerging as an alternative surgical approach due to its many technical strengths over laparoscopy, especially in complicated surgeries [12,13]. In addition, recently, a paradigm shift from multiport RAL to SPA RAL has emerged in various surgical fields due its advantages [14,15]. However, from a functional point of view, whether the wide range of robotic arm motions, precision of robotic instruments, and enhanced visualization of robotic cameras can positively affect the preservation of ovarian reserve remains unclear. 

Therefore, the purpose of this study was to compare SPA laparoscopy, the least invasive surgery, and SPA RAL, the most technically advanced surgery, for endometriosis with respect to the preservation of ovarian reserve and surgical outcomes. We also evaluated the clinical factors that could reduce ovarian function after surgery.

## 2. Materials and Methods

### 2.1. Inclusion and Exclusion Criteria

We retrospectively reviewed patients who were histologically diagnosed with endometriosis between January 2016 and December 2021 in the Department of Obstetrics and Gynecology at Samsung Medical Center, Seoul, South Korea. This study was approved by the Institutional Review Board (IRB) of the Sungkyunkwan University of Korea (IRB No. 2022-07-166). Patients who met the following criteria were eligible for this study: (1) those who had not reached menopause, (2) those who had endometrioma with or without extraovarian endometriosis including deep infiltrative endometriosis (DIE), (3) those who underwent conservative surgery using SPA laparoscopy or SPA RAL (conservative surgery was defined as the preservation of both ovaries), and (4) those with results of anti-Müllerian hormone (AMH) levels preoperatively, and at 2 weeks and 3 months postoperatively. We excluded patients who met one or more of the following criteria: (1) those who had previously undergone ovarian surgery and (2) those who underwent another gynecologic surgery (such as a hysterectomy or myomectomy) simultaneously.

### 2.2. Data Collection and Definition

The patients’ baseline characteristics, including age, parity, body mass index (BMI), ovarian tumor size, serum tumor marker (CA-125), disease stage measured by the 2021 American Association of Gynecologic Laparoscopists (AAGL) Endometriosis Classification [16], and history of abdominal surgery, were collected. The type of surgical approach (SPA laparoscopy or SPA RAL), operative time, perioperative complications, estimated blood loss (EBL), complexity of surgery (non-complex surgery or complex surgery), and duration of hospital stay were recorded for surgical outcomes. The serum AMH levels were recorded before surgery and at 2 weeks and 3 months after surgery to assess the ovarian reserve. Since the serum AMH levels are not affected by the menstrual cycle [17] or hormone treatments such as oral contraceptive pills or GnRH agonists [18], we used this marker to estimate the ovarian reserve. It is known that ovarian function decreases rapidly immediately after surgery and recovers gradually [19], but some studies report that there is no statistically significant recovery of ovarian reserve after 3 months postoperatively [20,21,22]. In addition, considering the follow-up loss of patients and the possibility of pregnancy during a long term follow-up period, the AMH check endpoint was determined as a relatively short period of 3 months. The serum AMH levels were measured by an enzyme-linked immunosorbent assay (ELSIA; Beckman Coulter AMH Gen II, Brea, CA, USA), following the manufacturer’s instruction. The detection limit of the assay was 0.08 ng/mL, and the intra- and inter-assay coefficients of variation were both <10%. The severity of endometriosis was retrospectively assessed using the AAGL classification based on surgical records and films of each surgery, and it was classified into mild endometriosis (early stage, AAGL stages I and II) or severe endometriosis (advanced stage, AAGL stages III and IV). Surgeries that were performed by ovarian cystectomy only, that included simple adhesiolysis which did not require the access of the retroperitoneal space, or that included fulguration alone, were categorized as non-complex conservative surgeries. Complex surgeries were defined as those with complex procedures, such as vesico-uterine or recto-vaginal space peritonectomy, urinary tract surgery, or bowel surgery due to DIE. To validate the accuracy of surgical complexity classification in our study, we analyzed the concordance with the AAGL surgical complexity classification [16]. The serum AMH level reduction rate was calculated using the following formula: rate of decline (%) = 100 × (AMH level before surgery–AMH level at 2 weeks or 3 months after surgery)/AMH level before surgery. The ovarian tumor size was defined as the maximal diameter of the tumor measured with an electronic caliper on preoperative ultrasonography, CT, or MRI images. If an ovarian tumor was bilateral or multiple, it was calculated as the sum of their respective sizes. The total operative time was defined as the time from skin incision to skin closure. EBL was calculated by the anesthesiology unit as the difference between the total amounts of suction and irrigation. Hemoglobin decrease was defined as the difference between the hemoglobin levels before surgery and on postoperative day one. The length of hospital stay was defined as the time from operation to discharge. Major complications were defined as bowel, bladder, or ureteral injuries requiring resection and re-anastomosis.

### 2.3. Surgical Methods

All surgical procedures were performed by a single surgeon (T.J. Kim) who had extensive experience in both SPA laparoscopy and RAL. There were no definite clinical criteria to determine the surgical approach (SPA laparoscopy or SPA RAL). However, prior to the surgery, detailed counseling was offered to every patient. During the counseling session, the patients were informed of the purpose, surgical methods, potential risks, benefits, cost, and insurance coverage of each surgical approach method. Subsequently, they were asked to choose their preferred method, and surgery was performed according to their decision. The counseling was non-directive.

Both laparoscopic surgeries and robotic surgeries were initially performed with a single-port approach. The surgical technique of SPA laparoscopy has been described previously [23]. Briefly, a small vertical transumbilical incision of 2.0 to 2.5 cm was made using the open Hasson technique. A single multichannel port (Glove PortTM; Nelis, Inc., Seoul, Republic of Korea) was inserted through the umbilicus. SPA laparoscopy was performed using a rigid, 30-degree, 5 mm laparoscope, 5 mm articulating laparoscopic grasper (RoticulatorTM; Covidien, Inc., Mansfield, MA, USA), and a conventional rigid straight laparoscopic instrument. RAL was performed using the da Vinci^®^ Xi system (Intuitive Surgical, Sunnyvale, CA, USA). The port placement was based on a single site platform at the umbilicus. If additional trocars were needed in a complex surgery, such as urinary tract and rectal surgery, one to three 8 mm robotic trocars were placed on the right and left sides of the umbilicus. A robotic single site curved monopolar hook, scissors, bipolar forceps, needle driver, or ProGraspTM forceps (in the case of multiport RAL) were used as appropriate. After port placement, pneumoperitoneum with carbon dioxide at 12 mmHg was established for both surgical approaches. First, we examined the pelvis and upper abdomen to assess the severity of adhesion and the extent of disease. Before initiating the stripping of the endometriotic cyst, adhesiolysis and mobilization of the ovary from adhesions with surrounding organs were performed. After identifying a cleavage plane between the cyst wall and the ovarian cortex, the ovary was pulled slowly and gently in the opposite direction using grasping forceps. We used bipolar coagulation as minimally as possible to minimize ovarian damage, only when its use was absolutely necessary. Instead, fibrin glue (TisseelTM; Baxter Healthcare Corp., Deerfield, IL, USA) and gauze compression were used for hemostasis. If the ovary required additional hemostasis, it was sutured with barbed suture material (3-0 StratafixTM; Ethicon, Johnson and Johnson, Somerville, NJ, USA). Patients with extraovarian endometriosis underwent additional surgeries (e.g., from simple excision of endometriosis spots to urinary tract and bowel surgery depending on the location and depth of the lesions). All ovarian endometriosis surgeries were performed with SPA laparoscopy and SPA RAL. For extraovarian endometriosis surgeries, additional trocars were inserted after ovarian surgery as needed depending on the complexity of surgery in both surgical methods (details of the final trocar setting are presented in Supplementary Table S5). The extracted endometriotic cysts and excised endometriotic lesions were placed into endopouch specimen retrieval bags and were removed through the umbilical hole. 

### 2.4. Statistical Analysis

All statistical analyses were performed using SPSS version 25.0 (IBM SPSS Statistics for Windows; IBM Corp., Armonk, NY, USA). Normality of the data was checked with the Shapiro–Wilk test. Data with normal distribution are presented as mean ± standard deviation (SD). The median (interquartile range, IQR) was used for data with non-normal distributions. Frequency distributions among categorical variables for the two surgical methods were compared using the chi-squared test or Fisher’s exact test. The concordance of surgical complexity between our classification and the AAGL system was assessed with a weighted kappa coefficient. Multiple linear regression analysis models were used to estimate the independent contributions of variables to the rate of decline in the AMH levels. A p-value < 0.05 was considered statistically significant.

## 3. Results

A total of 165 patients were included in the study. Of these, 87 (52.7%) patients underwent SPA laparoscopy, while 78 (47.3%) patients underwent RAL. The clinical characteristics of each group are summarized in Table 1. There were no significant differences in the age, BMI, prior abdominal surgery history, parity, ovarian cyst size, preoperative CA-125 level, and AMH level between the two groups. More patients with bilateral endometrioma received RAL. The severity of endometriosis (AAGL stage, details of the stage distribution are presented in Supplementary Table S1) and proportion of the patients with severe symptoms, such as dyschezia and infertility, were higher in the RAL group than in the SPA laparoscopy group. When the surgical complexity classification in our study was compared with the AAGL system, there was significant agreement in surgical complexity (Supplementary Table S2).

The surgical outcomes according to the two approaches are summarized in Table 2. The mean operative time was longer in the RAL group than in the SPA laparoscopy group (*p* < 0.001). Complex surgery was more frequently performed in the RAL group (83.3% for RAL vs. 50.6% for SPA, *p* = 0.045). Other surgical outcomes did not differ significantly between the two groups. In particular, the rates of AMH reduction at two weeks and three months postoperatively did not differ significantly between the two groups. 

**Figure 1:**
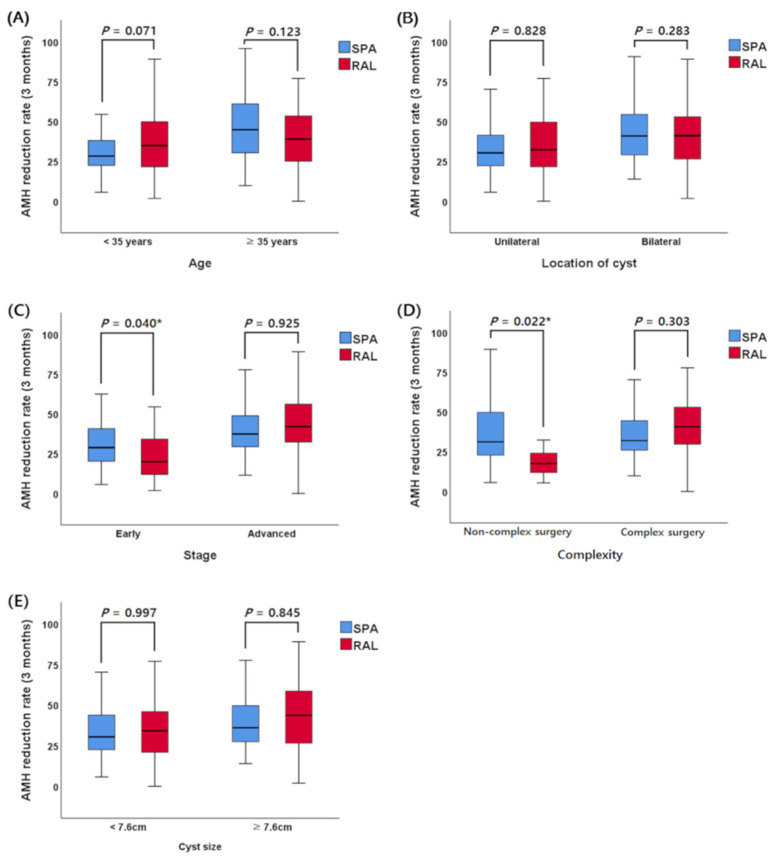
Anti-Müllerian hormone (AMH) reduction rates at 3 months postoperative according to surgical approach and each risk factor. (**A**) AMH reduction rate according to surgical approach and age, (**B**) AMH reduction rate according to surgical approach and location of cyst, (**C**) AMH reduction rate according to surgical approach and stage, (**D**) AMH reduction rate according to surgical approach and complexity of surgery, (**E**) AMH reduction rate according to surgical approach and cyst size.

In univariate and multivariate linear regression analyses of AMH reduction rates (Table 3), age (β = 0.686), ovarian cyst size (β = 1.483), location of ovarian cyst (β = 8.059), complexity of surgery (β = 4.670), and the severity of endometriosis (β = 9.953) were associated with the rate of decrease in AMH levels. However, the surgical approach was not associated with postoperative AMH reduction. When we compared the AMH reduction rates according to these risk factors (Supplementary Table S3), the decrease in ovarian function was more severe in patients with old age, bilateral endometrioma, severe endometriosis (advanced stage, AAGL stage III/IV), complex surgeries, and large ovarian cysts (*p* < 0.05). To determine which surgical method was beneficial for minimizing ovarian function damage, we also analyzed the postoperative AMH changes according to the surgical approach and each risk factor (Figure 1 and Supplementary Table S4). Reduction rates in the RAL group were smaller than those in the SPA laparoscopy group for patients with mild endometriosis (early stage, AAGL stage I/II) (AMH reduction rates at 3 months postoperative: RAL vs. SPA laparoscopy, 23.58 ± 14.98% vs. 33.51 ± 19.98%; *p* = 0.04). In addition, patients who underwent a non-complex surgery through robotic surgery showed less loss of ovarian function than those treated with SPA laparoscopy (AMH reduction rates at 3 months postoperative: RAL vs. SPA laparoscopy, 22.37 ± 17.49% vs. 37.89 ± 22.37%; *p* = 0.022). However, the difference in surgical method did not affect the change in ovarian function after surgery for other risk factors.

## 4. Discussion

This study was conducted to compare the ovarian reserve, represented by the serum AMH levels, and surgical outcomes after SPA laparoscopy and RAL for the management of patients with endometriosis. Furthermore, the factors affecting the reduction in ovarian function after endometriosis surgery were investigated. We found that although ovarian function decreased similarly after RAL and SPA laparoscopy in all patients, RAL had an advantage over SPA laparoscopy in terms of preserving ovarian function in patients with mild endometriosis and those who underwent non-complex surgery. We also found that age, tumor size, location of cyst, severity of disease, and complexity of surgery were associated with a decrease in ovarian function.

Complete resection of all visible lesions and restoration of the distorted pelvic anatomy are the treatment goals of endometriosis surgery to prevent recurrence and reduce pain [24]. Abesadze et al. have reported that successful removal of DIE in the posterior compartment of the deep pelvis reduces pain and recurrence and increases fertility rate [25,26]. In order to achieve this aim, endometriosis surgery can vary according to the severity of the disease from simple cystectomy of endometrioma to urinary tract and bowel surgery requiring advanced techniques. Laparoscopy is generally considered as the standard surgical approach. However, in complex cases with DIE in the bowel and urinary tract, it is difficult to perform surgery only with laparoscopy without conversion to laparotomy, even for experienced surgeons. Moreover, SPA laparoscopy is more challenging in these complicated cases due to its technical limitations [27]. In fact, the conversion rate to laparotomy during laparoscopic endometriosis surgery has been reported up to 36.4% (segmental bowel resection), depending on the complexity of surgery [28]. The development of an innovative robotic surgery system can provide magnified three-dimensional visualization, wristed instruments, and ergonomic positioning to the operator. This enables surgeons to perform high-level techniques that are difficult to implement in laparoscopy, such as deep pelvis dissection and extensive suturing, which can cause major complications during surgery. Some studies have been conducted to evaluate the benefit of RAL for endometriosis in comparison with laparoscopy [29,30,31,32,33,34]. Most studies have reported that RAL has no statistically proven benefit over laparoscopy in terms of perioperative outcomes. They even reported that RAL is a time-consuming and cost-ineffective method. However, it has been argued that RAL for endometriosis is expected to have potential technical benefits for severe endometriosis, although it has not yet been statistically proven. Its value should be highlighted, especially in complex surgeries, as it may lower the conversion rates to laparotomy. However, most studies so far have focused only on the technical advantages of RAL in endometriosis surgery, and there has been no discussion on the aspect of ovarian function preservation. 

In this study, the mean operative time in the RAL group (167.9 ± 63.5 min) was longer than that in the SPA laparoscopy group (93.7 ± 27.5 min, *p* < 0.001). However, the other values showed no significant differences between the two groups. Although docking time was added to the operative time, the longer operative time in the RAL group could be interpreted as due to the more extensive surgeries performed (complex surgery rates: RAL vs. SPA laparoscopy, 83.3% vs. 50.6%, *p* < 0.001) because there were many complicated cases, such as severe endometriosis, bilateral cysts, and patients with severe symptoms. Although more complex surgeries were performed in the RAL group, other surgical outcomes, including hemoglobin changes, hospital stay length, and complication rates, showed no significant differences. Many laparoscopic experts agree that robotic surgery is appropriate, especially for treating severe endometriosis, which requires difficult procedures. Of course, there are some limitations to concluding that RAL is superior to laparoscopy in complex endometriosis surgery based on the previous and present studies. To make a better decision about its value for complex endometriosis surgery, further studies such as case–control studies or randomized controlled trials are needed.

The majority of patients undergoing endometriosis surgery are reproductive-aged women desiring future fertility. Therefore, clinical outcomes (e.g., fertility rates or ovarian function preservation), as well as perioperative outcomes (e.g., operative time or hemoglobin changes), are important issues. It is known that patients with endometriosis have lower AMH levels at baseline than those with other benign ovarian tumors or healthy women due to the disease-induced chronic inflammatory response and immune modulation (3). Furthermore, thermal damage caused by an electric coagulator for hemostasis and non-meticulous stripping of the cystic capsule from normal ovarian tissue can cause loss of normal ovarian tissue and exacerbate ovarian function after surgery. Iwase et al. [35] found that AMH levels are decreased after laparoscopic ovarian cystectomy, regardless of histologic type. Such decreases are more profound in endometriomas than in other histologic types. Therefore, patients with endometriosis are inevitably vulnerable in terms of ovarian function preservation. Previous studies have revealed that various factors, including surgical approach, hemostatic method, cyst size, and cyst location, could influence the reduction in ovarian function after surgery [11,36,37]. In our analysis, similar to previous studies, the decrease in AMH levels was greater with increasing age, cyst size, and severity of endometriosis. In addition, patients with bilateral endometrioma or those who underwent complex surgery had a greater decrease in ovarian function. 

Regarding the surgical approach, the aforementioned technical advantages of robotic surgery can theoretically have a positive effect on the preservation of ovarian function. First, better visualization through the robotic camera can accurately detect the bleeding focus, thereby reducing the use of electric coagulation. It can also help identify the cleavage plane between the normal ovarian tissue and ovarian cystic capsule. Second, robotic instruments are more powerful and precise with a wider range of motion than laparoscopic instruments. Before initiating ovarian cystectomy, the robotic arms can better restore the normal anatomical positions of ovaries from their embedded positions in the posterior cul-de-sac, which often present with severe adhesions. During ovarian cystectomy, the robot can also be effective in stripping endometrioma capsules that are densely adherent to healthy ovarian tissues. Theoretically, this can reduce the loss of normal ovarian tissue during surgery. However, in our study, these theoretical advantages of RAL in terms of preserving ovarian function appeared only in certain patient groups, not in all patient groups. Whether the surgical action during laparoscopic ovarian cystectomy affects postoperative ovarian function is still debatable. Angioni et al. [11] have reported that SPA laparoscopy has a negative effect on ovarian reserve at three months after endometrioma surgery (AMH levels in SPA vs. conventional laparoscopy: 1.84 ± 1.51 vs. 2.11 ± 1.84, *p* < 0.01). They pointed out that rough surgical actions during SPA laparoscopy, caused by instrument conflicts and less ergonomic positions, could be possible causes. However, according to one randomized controlled study [38], reducing the number of laparoscopic ports during ovarian cystectomy does not affect ovarian function after surgery, even with endometrioma. For robotic surgery, to the best of our knowledge, only one study has evaluated the effect of robotic surgery on ovarian function preservation [39]. It reported that RAL was beneficial in bilateral endometrioma surgery compared to laparoscopic surgery, but this advantage did not appear in unilateral endometrioma surgery. However, that study only analyzed changes in AMH levels according to the surgical approach and location of endometrioma (unilateral vs. bilateral) without considering other clinical factors that could affect postoperative AMH levels. In the present study, we investigated whether RAL had advantages in ovarian function preservation compared to SPA laparoscopy for various clinical factors. As a result, the ovarian reserve was better preserved in patients with mild endometriosis and in patients who underwent non-complex surgery using RAL than in those who received SPA laparoscopy (Figure 1 and Supplementary Table S4). However, this advantage of RAL was not confirmed in relatively severe cases, such as patients with severe endometriosis and those who underwent complex surgery. The exact reason for this is difficult to explain due to the lack of studies. We carefully hypothesize that clinical factors in severe cases, such as the severity of disease or the extent of surgery, might have negative effects on the ovarian reserve sufficient to offset the positive effect of RAL on ovarian function [40]. Our results suggest that RAL might have advantages in terms of preserving the ovarian reserve when ovarian surgery is mainly performed without extensive adhesiolysis. However, there are several considerations in applying our results to clinical practice. This study only focused on the preservation of ovarian function with RAL, and it was beneficial in certain patient subgroups, but other clinical factors such as cost, operation time, and the accessibility of RAL were not considered. Therefore, it is important to comprehensively consider these factors and select the optimal surgical method for each patient. However, further studies are needed to demonstrate the exact role of RAL in severe cases. 

To the best of our knowledge, this is the first study to evaluate whether RAL has advantages in ovarian function preservation after endometriosis surgery according to impacting factors for postoperative ovarian reserve. Another strength of this study is that it compared the serial changes in AMH levels after endometriosis surgery between the robot group and the laparoscopy group for a relatively large number of patients. However, this study had some limitations. First, this was not a prospective, randomized clinical trial. Second, this study had a relatively small number of patients and a short follow-up period. Therefore, long-term clinical outcomes, such as pregnancy outcomes or menopause age, could not be assessed. Third, RAL is generally more expensive than SPA laparoscopy. However, this study did not consider cost-effectiveness, one of most important factors in determining the surgical method. Fourth, although all ovarian surgeries were performed with SPA RAL, additional 8 mm robotic trocars were used after ovarian surgery as needed in complex cases. Therefore, in a strict sense, there was no comparison between robot SPA laparoscopy and SPA laparoscopy in terms of final trocar settings (the final trocar settings of the RAL are shown in Supplementary Table S5). There is no doubt that SPA laparoscopy is the most technically challenging among laparoscopic procedures. However, whether SPA laparoscopy adversely affects ovarian reserve preservation after endometriosis surgery, as compared to conventional laparoscopy, has not yet been clarified [11,38]. Despite some differences in the number of trocars used in RAL and SPA laparoscopy, we think that this study is meaningful in that it compares the use of a robotic system and laparoscopy.

In conclusion, RAL may have an advantage in terms of preserving ovarian reserve in certain patient groups, such as patients with mild endometriosis and those who underwent non-complex surgery. Furthermore, RAL is a very attractive surgical approach for patients with complex endometriosis. Further studies are needed to support our initial findings. 

## Figures and Tables

**Table 1:** Characteristics of patients who underwent SPA laparoscopy or RAL.

Clinical Characteristics	Total	SPA	Robot	*p*-Value
	(n = 165)	(n = 87)	(n = 78)	
Age (years)	33.3 ± 6.9	33.1 ± 7.09	33.5 ± 6.92	0.666
BMI (kg/m^2^)	21.3 ± 3.0	21.2 ± 2.92	21.4 ± 3.02	0.674
Abdominal surgical history	28 (17.0%)	12 (13.8%)	16 (20.5%)	0.301
Parity				0.760
Nulliparous	123 (74.5%)	64 (73.6%)	59 (75.6%)	
Parous	42 (25.5%)	23 (26.4%)	19 (24.4%)	
Symptoms				
Dysmenorrhea	133 (80.6%)	71 (81.6%)	62 (79.5%)	0.844
Dyschezia	26 (15.8%)	7 (8.0%)	19 (24.4%)	0.005 *
Dyspareunia	16 (9.7%)	5 (5.7%)	11 (14.1%)	0.112
Pelvic pain	53 (32.1%)	24 (27.6%)	29 (37.2%)	0.242
Infertility	7 (4.2%)	1 (1.1%)	6 (7.7%)	0.037 *
Location of ovarian cyst				0.002 *
Unilateral	99 (60.0%)	62 (71.3%)	37 (47.4%)	
Bilateral	65 (40.0%)	25 (28.7%)	41 (52.6%)	
CA-125 level (IU/mL)	32.6 ± 30.5	30.3 ± 33.6	34.8 ± 27.1	0.397
Preoperative AMH level (ng/mL)	3.02 ± 2.50	3.18 ± 2.63	2.85 ± 2.35	0.409
Ovarian cyst size (cm)	7.6 ± 2.9	7.4 ± 2.5	7.9 ± 3.4	0.258
AAGL stage				<0.001 *
stage I/II	73 (44.2%)	51 (58.6%)	22 (28.2%)	
stage III/IV	92 (55.8%)	36 (41.4%)	56 (71.8%)	

Abbreviations: SPA: single-port access; RAL: robot assisted laparoscopy; BMI: body mass index; AMH: anti-Müllerian hormone; AAGL: American Association of Gynecologic Laparoscopists. Values are given as mean ± standard deviation or number (percentage). * *p* < 0.05.

**Table 2:** Surgical outcomes of SPA laparoscopy or RAL.

Clinical Characteristics	Total	SPA	Robot	*p*-Value
	(n = 165)	(n = 87)	(n = 78)	
Operative time (min)	128.8 ± 60.6	93.7 ± 27.5	167.9 ± 63.5	<0.001 *
EBL (mL)	100 (50–150)	100 (50–125)	100 (50–150)	0.356
Hospital stay (days)	2 (2–2)	2 (2–2)	2 (2–3)	0.391
Hb decrease (g/dL)	1.8 ± 1.0	1.7 ± 1.0	1.9 ± 1.0	0.369
Complexity of surgery				<0.001 *
Non-complex surgery	56 (33.9%)	43 (49.4%)	13 (16.7%)	
Complex surgery	109 (66.1%)	44 (50.6%)	65 (83.3%)	
Major complication	0	0	0	n/a
Conversion to laparotomy	0	0	0	n/a
Serum AMH level (ng/mL)				
Pre-op	3.02 ± 2.50	3.18 ± 2.63	2.85 ± 2.35	0.409
Post-op 2 weeks	1.67 ± 1.59	1.83 ± 1.68	1.50 ± 1.48	0.191
Post-op 3 months	1.96 ± 1.75	2.13 ± 1.86	1.78 ± 1.61	0.195
AMH reduction rate (%)				
Pre-op–Post-op 2 weeks	48.6 ± 18.4	47.8 ± 19.3	49.3 ± 17.6	0.613
Pre-op–Post-op 3 months	37.4 ± 20.1	37.3 ± 20.4	37.5 ± 19.8	0.938

Abbreviations: SPA, single-port access; RAL, robot assisted laparoscopy; EBL, estimated blood loss; Hb, hemoglobin; AMH, anti-Müllerian hormone; n/a, not available; op, operation. Values are given as mean ± standard deviation, median (interquartile range), or number (percentage). * *p* < 0.05.

**Table 3:** Simple and multiple linear regression analyses for the rate of decrease in AMH levels recorded at 2 weeks and 3 months postoperative.

	**AMH Reduction Rate at 2 Weeks**							
**Clinical factors**	**Simple regression analysis**				**Multiple regression analysis**			
	**R^2^**	**Β**	**SE**	***p***	**R^2^**	**β**	**SE**	***p***
Age	0.072	0.708	0.199	<0.001 *	0.117	0.696	0.191	<0.001 *
BMI	0.010	0.617	0.485	0.205	-	-	-	-
Size	0.034	1.162	0.483	0.017 *	0.117	1.047	0.459	0.024 *
Bilaterality ^a^	0.041	7.633	2.884	0.009 *	-	-	-	-
Stage ^b^	0.038	7.228	2.850	<0.001 *	0.117	9.176	2.711	0.001 *
Surgical method ^c^	0.002	1.462	2.888	0.613	-	-	-	-
Complexity ^d^	0.024	6.084	3.010	0.045 *	-	-	-	-
	**AMH Reduction Rate at 3 Months**							
**Clinical factors**	**Simple regression analysis**				**Multiple regression analysis**			
	**R^2^**	**β**	**SE**	***p***	**R^2^**	**β**	**SE**	***p***
Age	0.057	0.686	0.219	0.002 *	0.126	0.686	0.207	0.001 *
BMI	0.005	0.465	0.529	0.381	-	-	-	-
Size	0.057	1.622	0.519	0.002 *	0.126	1.483	0.496	0.003 *
Bilaterality ^a^	0.039	8.059	3.141	0.011 *	-	-	-	-
Stage ^b^	0.043	8.326	3.093	<0.001 *	0.126	9.953	2.934	0.001 *
Surgical method ^c^	0.005	5.947	4.156	0.154	-	-	-	-
Complexity ^d^	0.012	4.670	3.295	0.158	-	-	-	-

Abbreviations: AMH, anti-Müllerian hormone; R^2^, coefficient of determination; β, regression coefficient; SE, standard error; BMI, body mass index. ^a^ Unilateral is the reference category. ^b^ Early stage (stage I/II) is the reference category. ^c^ SPA is the reference category. ^d^ Non-complex conservative surgery is the reference category. * *p* < 0.05.

## Data Availability

The data presented in this study are available on request from the corresponding author.

## Supplementary Materials

The following supporting information can be downloaded at: https://www.mdpi.com/article/10.3390/jcm12144673/s1, Table S1: The AAGL stage distribution of patients; Table S2: Concordance between the radicality of surgery in our study and the surgical complexity by AAGL classification; Table S3: AMH level changes and AMH reduction rates according to risk factors; Table S4: AMH reduction rates according to surgical method and risk factors. Table S5. The final trocar setting details of the RAL.
