# Supplementary material for: Does Robot Assisted Laparoscopy (RAL) Have an Advantage in Preservation of Ovarian Reserve in Endometriosis Surgery? Comparison of Single-Port Access (SPA) RAL and SPA Laparoscopy
Source: J Clin Med. 2023 Jul 14;12(14):4673. doi: 10.3390/jcm12144673 (PMC10380253; doi:10.3390/jcm12144673)
Supplement: Supplementary file 1 [file jcm-12-04673-s001.zip › jcm-2474189-supplementary.pdf]

Does Robot Assisted Laparoscopy (RAL) Have an Advantage in Preservation of Ovarian Reserve in Endometriosis Surgery? Comparison of RAL and Single-Port Access (SPA) Laparoscopy

Jun-Hyeok Kang[1†], Chi-Son Chang[2†], Joseph J. Noh[3], Jungeun Jeon[3], Ji-Hee Jung[3], Tae-Joong Kim[3]

Supplementary Table S1. The AAGL stage distribution of patients

AAGL stage				
**Early stage (*n*=73)**		**Advanced stage (*n*=92)**		**Total**
**I**	**II**	**III**	**IV**	
3 (1.8)	70 (42.4)	62 (37.5)	30 (18.2)	165 (100)

AAGL, American Association of Gynecologic Laparoscopists.

Values are given number (percentage).

Supplementary Table S2. Concordance between the radicality of surgery in our study and the surgical complexity by AAGL classification

Complexity of surgery	Non-complex surgery	Complex surgery	Total	κ = 1.000
**AAGL level A and B**	56 (33.9)	0 (0)	56 (33.9)	
**AAGL level C and D**	0 (0)	109 (66.1)	109 (66.1)	
**Total**	56 (39.9)	109 (66.1)	165 (100)	

AAGL, American Association of Gynecologic Laparoscopists.

Non-complex surgeries are defined as those with simple procedures such as ovarian cystectomy only or simple adhesiolysis which did not require the access of the retroperitoneal space, or fulguration.

Complex surgeries are defined as those with complex procedures such as vesico-uterine or recto-vaginal space peritonectomy, urinary tract surgery, or bowel surgery.

Values are given number (percentage).

Supplementary Table S3. AMH level change and AMH reduction rate according to risk factors.

**Age**	**<35 years (*n* = 98)**	**≥35 years (*n* = 67)**	***p***
Serum AMH level (ng/mL)			
Pre-op	4.05 ± 2.48	1.52 ± 1.63	<0.001*
Post-op 2 weeks	2.34 ± 1.68	0.69 ± 0.72	<0.001*
Post-op 3 months	2.70 ± 1.81	0.89 ± 0.88	<0.001*
AMH reduction rate (%)			
Post-op – Post-op 2 weeks	43.87 ± 16.83	55.44 ±18.74	<0.001*
Post-op – Post-op 3 months	33.34 ± 16.87	43.29 ± 22.95	0.002
**Location of cyst**	**Unilateral (*n* = 99)**	**Bilateral (*n* = 66)**	***p***
Serum AMH level (ng/mL)			
Pre-op	3.37 ± 2.58	2.48 ± 2.29	0.025*
Post-op 2 weeks	1.94 ± 1.63	1.26 ± 1.45	0.006*
Post-op 3 months	2.25 ± 1.74	1.52 ± 1.68	0.008*
AMH reduction rate (%)			
Post-op – Post-op 2 weeks	45.51 ± 18.40	53.14 ± 17.76	0.009*
Post-op – Post-op 3 months	34.15 ± 18.99	42.21 ± 20.88	0.011*
**Severity of disease**	**Early stage (*n* = 73)**	**Advanced stage (*n* = 92)**	***p***
Serum AMH level (ng/mL)			
Pre-op	3.42 ± 2.73	2.70 ± 2.26	0.071
Post-op 2 weeks	2.11 ± 1.83	1.32 ± 1.28	0.002*
Post-op 3 months	2.44 ± 1.95	1.58 ± 1.47	0.002*
AMH reduction rate (%)			
Post-op – Post-op 2 weeks	42.38 ± 18.88	53.47 ± 16.68	<0.001*
Post-op – Post-op 3 months	30.51 ± 19.07	42.82 ± 19.30	<0.001*
**Complexity of surgery**	**Non-complex (*n* = 56)**	**Complex (*n* = 109)**	***p***
Serum AMH level (ng/mL)			
Pre-op	3.99 ± 2.83	2.52 ± 2.16	<0.001*
Post-op 2 weeks	2.41 ± 1.92	1.29 ± 1.23	<0.001*
Post-op 3 months	2.77 ± 2.05	1.55 ± 1.41	<0.001*
AMH reduction rate (%)			
Post-op – Post-op 2 weeks	44.55 ± 21.03	50.63 ± 16.75	0.045*
Post-op – Post-op. 3 months	34.29 ± 21.68	38.96 ± 19.14	0.158
**Cyst size**	**<7.6 cm (*n* = 91)**	**≥7.6 cm (*n* = 74)**	***p***
Serum AMH level (ng/mL)			
Pre-op	3.07 ± 2.59	2.95 ± 2.39	0.772
Post-op 2 weeks	1.72 ± 1.51	1.62 ± 1.69	0.688
Post-op 3 months	2.05 ± 1.66	1.86 ± 1.86	0.499
AMH reduction rate (%)			
Post-op – Post-op 2 weeks	45.9 ± 17.8	51.8 ± 18.9	0.042*
Post-op – Post-op 3 months	33.6 ± 18.4	42.0 ± 21.2	0.007*

AMH, anti-Müllerian hormone; OP, operation.

Values are given as mean ± standard deviation.

Supplementary Table S4. AMH reduction rate according to surgical method and risk factors

**Age**	**<35 years (*n* = 98)**			**≥35 years (*n* = 67)**		
**AMH reduction rate**	**SPA (*n* = 52)**	**Robot (*n* = 46)**	***p***	**SPA (*n* = 35)**	**Robot (*n* = 32)**	***p***
2 weeks (%)	41.16 ± 14.82	46.9 ± 18.53	0.091	57.85 ± 20.97	52.81 ± 15.85	0.274
3 months (%)	30.45 ± 12.70	36.60 ± 20.23	0.071	47.38 ± 25.33	38.80 ± 19.42	0.123
**Location of cyst**	**Unilateral (*n* = 99)**			**Bilateral (n=66)**		
**AMH reduction rate**	**SPA (*n* = 62)**	**Robot (*n* = 37)**	***p***	**SPA(*n* = 62)**	**Robot (*n* = 37)**	***p***
2 weeks (%)	44.54 ± 19.40	47.14 ± 16.71	0.501	56.13 ± 16.62	51.32 ± 18.38	0.289
3 months (%)	33.83 ± 19.09	34.93 ± 19.08	0.828	45.77 ± 21.68	40.04 ± 20.34	0.283
**Severity of disease**	**Early (*n* = 73)**			**Advanced (*n* = 92)**		
**AMH reduction rate**	**SPA (*n* = 51)**	**Robot (*n* = 22)**	***p***	**SPA (*n* = 36)**	**Robot (*n* = 56)**	***p***
2 weeks (%)	44.31 ± 19.70	37.92 ± 16.37	0.187	52.92 ± 17.74	53.82 ± 16.12	0.803
3 months (%)	33.51 ± 19.98	23.58 ± 14.98	0.040*	42.57 ± 20.25	42.97 ± 18.86	0.925
**Complexity of surgery**	**Non-complex (*n* = 56)**			**Complex (n=109)**		
**AMH reduction rate**	**SPA (*n* = 43)**	**Robot (*n* = 13)**	***p***	**SPA (*n* = 44)**	**Robot (*n* = 65)**	***p***
2 weeks (%)	47.16 ± 21.38	35.88 ± 17.91	0.090	48.57 ± 17.22	52.03 ± 16.41	0.297
3 months (%)	37.89 ± 22.37	22.37 ± 17.49	0.022*	36.64 ± 19.42	40.53 ± 18.95	0.303
**Cyst size**	**<7.6 cm (*n* = 91)**			**≥7.6 cm (n=74)**		
**AMH reduction rate**	**SPA (*n* = 47)**	**Robot (*n* = 44)**	***p***	**SPA (*n* = 40)**	**Robot (*n* = 34)**	***p***
2 weeks (%)	43.56 ± 18.15	48.47 ± 17.31	0.191	52.94 ± 19.58	50.46 ± 18.21	0.576
3 months (%)	33.60 ± 18.87	33.61 ± 18.21	0.997	41.56 ± 21.66	42.54 ± 20.91	0.845

AMH, anti-Müllerian hormone; SPA, single-port access; RAL, robot assisted laparoscopy.

Values are given as mean ± standard deviation.

Supplementary Table S5. The final trocar setting details of the RAL

	RAL (*n*=78)		Total	SPA (*n*=87)		Total
	Single site (*n*=71)	Multiport (*n*=7)		Single port (n=87)	Multiport (*n*=0)	
AAGL stage						
Early stage	22 (100)	0 (0)	22 (100)	51 (100)	0 (0)	51 (100)
Advanced stage	49 (87.5)	7 (12.5)	56 (100)	36 (100)	0 (0)	36 (100)
Complexity of surgery						
Non-complex surgery	13 (100)	0 (0)	13 (100)	43 (100)	0 (0)	43 (100)
Complex surgery	58 (89.2)	7 (10.8)	65 (100)	44 (100)	0 (0)	44 (100)

AAGL, American Association of Gynecologic Laparoscopists; RAL, Robot Assisted Laparoscopy. Values are given number (percentage).
